# Prognostic Value of CD8+ Lymphocytes in Hepatocellular Carcinoma and Perineoplastic Parenchyma Assessed by Interface Density Profiles in Liver Resection Samples

**DOI:** 10.3390/cancers15020366

**Published:** 2023-01-05

**Authors:** Rokas Stulpinas, Dovile Zilenaite-Petrulaitiene, Allan Rasmusson, Aiste Gulla, Agne Grigonyte, Kestutis Strupas, Arvydas Laurinavicius

**Affiliations:** 1 Faculty of Medicine, Institute of Biomedical Sciences, Department of Pathology, Forensic Medicine and Pharmacology, Vilnius University, 03101 Vilnius, Lithuania; 2 National Center of Pathology, Affiliate of Vilnius University Hospital Santaros Clinics, 08406 Vilnius, Lithuania; 3 Faculty of Medicine, Institute of Clinical Medicine, Vilnius University, 03101 Vilnius, Lithuania; 4 Department of Surgery, Memorial Sloan Kettering Cancer Center, New York, NY 10065, USA; 5 Faculty of Medicine, Vilnius University, 03101 Vilnius, Lithuania

**Keywords:** CD8, hepatocellular carcinoma, immune response, tumor-infiltrating lymphocytes, liver-infiltrating lymphocytes, digital image analysis, immunohistochemistry, computational pathology

## Abstract

**Simple Summary:**

In this study, we present the overall survival and recurrence-free survival models based on CD8 profiles in hepatocellular carcinoma (HCC) and peritumoral liver tissue, including other clinical and pathological variables. We extracted CD8 distribution profiles from the digitized immunohistochemistry slides containing the HCC-stroma interface and the perineoplastic liver parenchyma-stroma interface using the computational method of interface zone immunogradient. The prognostic value of the CD8+ cell spatial distribution indicators from both interfaces as well as clinical, laboratory, and pathology data was assessed. A three-tier prognostic scoring system is proposed to predict overall survival in patients with HCC.

**Abstract:**

Hepatocellular carcinoma (HCC) often emerges in the setting of long-standing inflammatory liver disease. CD8 lymphocytes are involved in both the antitumoral response and hepatocyte damage in the remaining parenchyma. We investigated the dual role of CD8 lymphocytes by assessing density profiles at the interfaces of both HCC and perineoplastic liver parenchyma with surrounding stroma in whole-slide immunohistochemistry images of surgical resection samples. We applied a hexagonal grid-based digital image analysis method to sample the interface zones and compute the CD8 density profiles within them. The prognostic value of the indicators was explored in the context of clinicopathological, peripheral blood testing, and surgery data. Independent predictors of worse OS were a low standard deviation of CD8+ density along the tumor edge, high mean CD8+ density within the epithelial aspect of the perineoplastic liver-stroma interface, longer duration of surgery, a higher level of aspartate transaminase (AST), and a higher basophil count in the peripheral blood. A combined score, derived from these five independent predictors, enabled risk stratification of the patients into three prognostic categories with a 5-year OS probability of 76%, 40%, and 8%. Independent predictors of longer RFS were stage pT1, shorter duration of surgery, larger tumor size, wider tumor-free margin, and higher mean CD8+ density in the epithelial aspect of the tumor-stroma interface. We conclude that (1) our computational models reveal independent and opposite prognostic impacts of CD8+ cell densities at the interfaces of the malignant and non-malignant epithelium interfaces with the surrounding stroma; and (2) together with pathology, surgery, and laboratory data, comprehensive prognostic models can be constructed to predict patient outcomes after liver resection due to HCC.

## 1. Introduction

Primary liver cancer is the third most common cause of cancer-related deaths in the world, with hepatocellular carcinoma (HCC) comprising up to 85% of cases [1]. Although general risk factors for HCC development are well known and surveillance guidelines have been established, the diagnosis of an individual HCC patient is complicated by both the asymptomatic nature of early-stage HCC [2] and the fact that 90% of HCC cases develop in the background of cirrhosis caused by chronic inflammatory liver disease [3]. This persistent inflammatory environment is mostly due to viral infections (HBV or HCV), autoimmune hepatitis, nonalcoholic fatty liver disease, excessive alcohol consumption, or other toxins [3,4]. It has been suggested that a persistent and inefficient immune system response is the cause of both inflammatory liver damage [5] and carcinogenesis [4,5]. In practice, cirrhosis obscures the HCC to the point where therapeutic options become limited [6].

Poor overall survival (OS) and high recurrence rates for patients with HCC emphasize the need for reliable prognostic models to avoid over- or undertreatment [7,8]. Furthermore, the underlying irreversible damage to the liver parenchyma limits established cancer-directed therapies and clinical trials because conventional chemo- and radiotherapy modes are either contraindicated or ineffective in cirrhotic patients [6]. Consequently, the standard potentially curative treatments for HCC are liver transplantation, surgical resection, and local ablative therapy [9]. However, HCC reoccurs in more than 50% of patients in 3 years [10] and in up to 70%, 5 years after surgical resection [11]. New treatment opportunities such as immune checkpoint inhibitors, inhibitory cytokine blockade, oncolytic viruses, adaptive cell therapies, and immunotherapeutic vaccines [12] have emerged; however, the effect of HCC immunotherapy is rather patient-specific, further raising the importance of robust biomarkers of HCC progression.

Assays to estimate the risk of developing and to predict the outcomes of HCC are actively sought. Serum alpha-fetoprotein (AFP) has been used as a biomarker of HCC for over half a century but remains controversial, as many experts consider it nonspecific [13]. Furthermore, most other biomarkers lack informative power if used individually as outcome predictors. Stratification systems based on multiple features, such as the Okuda, Barcelona Clinic Liver Cancer (BCLC), the Italian (CLIP), and French classifications, have been in use for decades [14]. These scoring schemes consider both the tumor properties and the remaining liver function indicators for a comprehensive assessment of liver disease. For example, the BALAD model measures bilirubin, albumin, two forms of alpha-fetoprotein (AFP-L3 and total AFP), and des-γ-carboxyprothrombin from a single blood serum sample [15]. Albumin and bilirubin provide estimates of the remaining liver function, while the combination of other indicators represents the stage and progression of the tumor. The combined results allowed the prediction of HCC patient survival with a hazard ratio of 1.43 per increase of 1 BALAD score [16]. In 2014, a diagnostic iteration of the latter model, named GALAD [13], replaced bilirubin and albumin with the patient’s age and sex to predict the presence of HCC; the model score appears to be proportional to the tumor cell mass and performs substantially better than any individual biomarker. Several years later, Sposito et al. proposed a simple scoring scheme based on routine clinical data for a robust stratification of patients with HCC eligible for resection; the model included an end-stage liver disease (MELD) score, HCV infection, the number of tumor lesions, their maximum size, and signs of portal invasion [10].

A novel set of biomarkers for the prognosis of HCC is also gaining the spotlight in the context of advances in immunotherapy. These indicators focus primarily on the distribution and populations of immune cells within the tumor microenvironment (TME) and the non-tumor microenvironment (NTME) [7,17]. However, the assessment of the immune response in liver tissue is complicated by a ‘dual’ pathology of HCC: a beneficial antitumoral response is conveyed by cytotoxic CD8+ T cells [18], which may have an opposite detrimental effect on the remaining functional liver parenchyma. In particular, in patients with HCC with known HBV or HCV infection, hepatocyte injury is caused by an immune response (involving CD8+ lymphocytes) rather than direct viral damage [4]. One can hypothesize that tumor-infiltrating lymphocytes (TILs) in HCC and non-tumor infiltrating lymphocytes (NILs) in the surrounding liver parenchyma may have independent effects on patient outcomes, hence the precise spatial distribution analysis of immune cells is required.

The role of TILs/NILs in HCC has long been under investigation (mainly based on immunohistochemistry studies); however, their significance in disease progression remains controversial. In 2004, Ikeguchi et al. [19] reported a lower average density of manually counted CD8+ cells in HCC tissue compared to surrounding non-cancerous hepatic lobules. They detected neither a correlation between the individual sample densities of TILs and NILs nor any prognostic significance of CD8+ cell densities. Ramzan et al. demonstrated that high levels of TILs reflect inflammatory conditions and contribute to tumorigenesis and the recurrence of HCC [20]. Abdou et al. found no effect of TILs on patient survival or recurrence [21]. Other studies reported that high levels of TILs were associated with better OS and disease-free survival (DFS) [8,22,23]. In 2013, An et al. [24] published a study of CD4+ and CD8+ cell infiltrates in HCC and perineoplastic liver tissue microarrays with the semiquantitative visual assessment of hotspots. They found an increased number of CD8+ cells in smaller tumors (≤5 cm) and, independently, in the peritumoral liver parenchyma in cases with chronic hepatitis and cirrhosis; unfortunately, the patient outcomes were not assessed in this study. In their meta-analysis, Xu et al. [22] concluded that OS was significantly longer in cases with high CD8+ TIL densities at the edge of the tumor, while patients with low TIL density had a more advanced TNM stage and a larger tumor size; however, no significance of lymphocytes infiltrating peritumoral tissues was established. It was noted that the vast majority of the reviewed studies were conducted in Asia where HBV is still the most prevalent etiological factor; therefore, more evidence needs to be collected from non-Asian populations to assess the significance of TILs in other HCC subgroups [22].

The well-established digital immunohistochemistry-based method Immunoscore assessing the host anti-tumoral response quantifies two (CD3 and CD8) lymphocyte populations at both the invasive margin (IM) and at the core of the tumor (CT) [25,26]. The method was originally proposed to predict a recurrence of stage II/III colon cancer and was later applied to other types of solid cancer [27]. In particular, Gabrielson et al. [28] demonstrated that patients with a high density of CD3+ and CD8+ cells in one or both regions of interest (IM or CT) and their corresponding ‘immunoscores’ were associated with a lower rate of HCC recurrence and longer relapse-free survival (RFS). Sun et al. [8] argued that although Immunoscore was closely related to the outcome of patients with HCC, it was not an optimal prognostic biomarker, since they observed that CD8+ density in the center of the tumor has the highest prognostic impact for both DFS and OS by Cox multivariate regression analysis. Liu et al. [29] recently reported a comprehensive HCC immunohistochemical study of 14 immune cell subtypes in three representative images captured in the tumor (T) and peritumoral (P) regions to quantify immune cell densities using a computer-automated method. They developed and validated a nine-factor IHC classifier (CD27_T_, CD57_T_, CD57_P_, CD45RA_P_, CD45RO_T_, CD66b_T_, CD68_P_, CXCR5_P_, and PD-1_T_) to predict recurrence in patients with early-stage HCC, but neither CD3 nor CD8 entered the final model. These findings on the significance of the spatial arrangement of immune cells in HCC warrant the study exploring the CD8+ distribution across the tumor-stroma interface in both malignant and benign (peritumoral) regions of the resected liver tissue.

Many studies have shown the potential of digital image analysis (DIA) and computational feature extraction tools from pathology images [30,31]. Automated, data-driven, and operator-independent methods can extract subvisual features that represent tissue properties and can be integrated into predictive models along with clinical and pathological parameters [32,33,34,35]. In liver pathology, DIA has also been proven to be a useful tool in both neoplastic and non-neoplastic settings [36,37,38]. Liao et al. used an automated computational deep learning model to extract prognostic characteristics from HCC and surrounding normal tissue from publicly available hematoxylin-eosin (H&E) whole slide images (WSI) [39]. Although this provided quantitative pathology features that were used to successfully diagnose HCC and predict the clinical outcomes of patients, the study was limited by the lack of important clinical information and serum biomarkers in a large proportion of the patients, and by a semiquantitative method of lymphocyte infiltration analysis. Similarly, most research is focused on applying convolutional neural networks (CNNs) to automatically extract features in H&E-stained liver tissue samples [40], while efforts with regard to the IHC-based computational assessment of TILs and NILs are lacking.

In this study, we present the OS and RFS models based on CD8 profiles in the tumor and peritumoral liver tissue, including other variables. We extracted CD8 distribution profiles from the HCC-stroma interface and from the perineoplastic liver parenchyma-stroma interface using the computational method of interface zone immunogradient [41]. The prognostic value of the CD8+ cell spatial distribution indicators from both interfaces as well as clinical, laboratory, and pathology data was assessed. A three-tier prognostic scoring system is proposed to predict OS in patients with HCC.

## 2. Materials and Methods

### 2.1. Study Population

The retrospective cohort used in this study included 106 patients (24 females and 82 males) who underwent liver resection for HCC in Vilnius University Hospital Santaros Clinics (Vilnius, Lithuania) from 2007 to 2020; the resection samples were analyzed at the National Center of Pathology (Vilnius, Lithuania). Clinical, laboratory, and pathology data were retrospectively collected according to the approval of the Vilnius Regional Biomedical Research Ethics Committee (permit number 2021/6-1354-843), which waived the requirement of individual informed consent according to the International Ethical Guidelines for Health-related Research Involving Humans [42].

The median time of OS was 39 (range 1–152) months calculated from the date of the first pathologically proven diagnosis of HCC, and for RFS it was 25 (range 1–174) months to the date of the first documented statement of HCC relapse. A summary of patient and tumor characteristics is presented in Table 1, and the results of preoperative blood tests are listed in Table 2.

### 2.2. Immunohistochemistry

**Figure 1:**
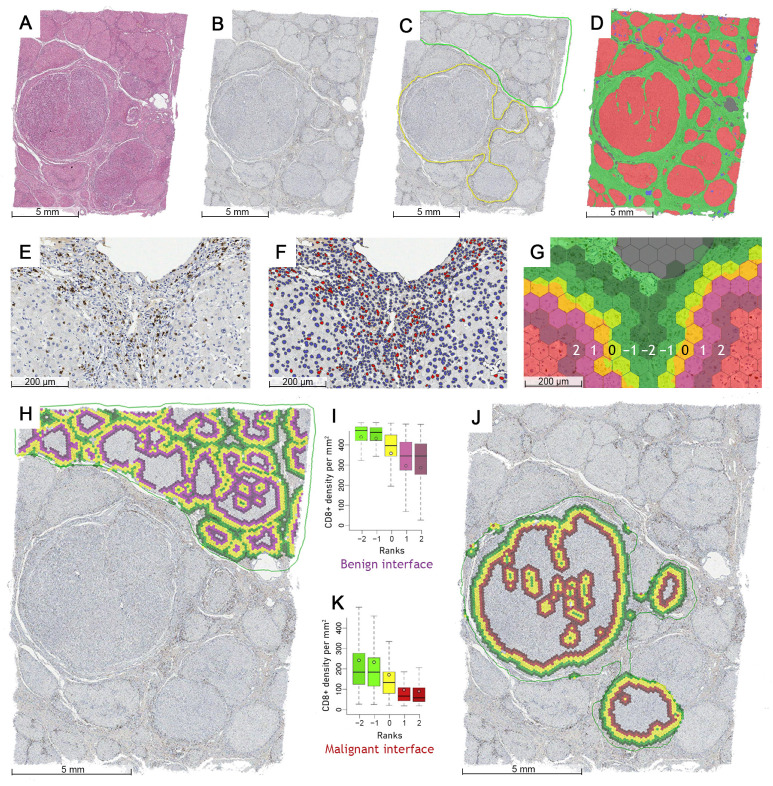
Study workflow (stroma interfaces with neoplastic (HCC) tissue and non-malignant liver parenchyma sampled to assess CD8+ cell density profiles): (**A**) low magnification view of the whole H&E stained tissue sample containing HCC nodules and the surrounding cirrhotic liver parenchyma; (**B**) a matching CD8 immunohistochemistry slide that was further used for analysis (see panel E and Supplementary Figure S1A,B for higher magnification); (**C**) a sample of manually annotated HCC and non-neoplastic liver areas, avoiding ambiguous or artifact-containing zones; (**D**) HALO^®^AI epithelium versus stroma segmentation: both malignant and non-malignant hepatocytes—red, stroma—green, debris and artifacts—blue, background—gray; (**E**) magnified detection zone on a CD8 slide, positive cytotoxic T lymphocytes—brown cytoplasmic staining; (**F**) Multiplex IHC^®^ nuclei segmentation masks, CD8 positive cells—red, CD8 negative cells—blue; (**G**) identification of the epithelial edge (yellow hexagons, rank 0), epithelial aspect (red hexagons, ranks 1–2) and stromal aspect (green hexagons, ranks −1 and −2) of the interface zone (ranks from −2 to 2) based on a hexagonal grid; (**H**) the benign (liver) interface zone, epithelial aspect (i.e., liver parenchyma)—purple, epithelial edge—yellow, stromal aspect—green; (**I**) an example boxplot showing a decrease of average CD8+ cell density per rank towards the epithelial aspect of the benign interface zone; (**J**) the malignant (HCC) interface zone, epithelial aspect (i.e., HCC)—red, epithelial edge—yellow, stromal aspect—green; (**K**) an example boxplot showing the decrease of average CD8+ cell density per rank towards the epithelial aspect of the malignant interface zone.

All available archived slides stained with hematoxylin and eosin were reviewed by a pathologist (RS) to select the most informative formalin-fixed paraffin-embedded (FFPE) block that contains both non-necrotic HCC tissue (N = 106) and the surrounding liver parenchyma if present (N = 100, 94.3%) (Figure 1A). The samples were cut to a thickness of 3 µm and mounted on positively charged slides and stained for CD8 antibody (Dako, clone C8/144B, dilution 1:100, Denmark) on a Roche Ventana BenchMark ULTRA automated stainer (Ventana Medical Systems, Oro Valley, AZ, USA), using the ultraView Universal DAB Detection kit (Ventana Medical Systems, Oro Valley, AZ, USA).

### 2.3. Digital Image Analysis and Indicator Extraction

All slides stained with H&E and CD8 were digitized (Figure 1A,B) at 20× magnification (0.5 µm per pixel) using an Aperio^®^ AT2 DX scanner (Leica Aperio Technologies, Vista, CA, USA). A pathologist (RS) then manually annotated the largest available continuous malignant and non-malignant area on each slide while avoiding ambiguous or artifact-containing zones (Figure 1C), as the HALO^®^ AI (Indica Labs, Albuquerque, NM, USA) classifier was not sufficient to fully discriminate between the well-differentiated HCC and reactive or dysplastic epithelium in some of the hematoxylin counterstained IHC slides. Thus, the AI system was trained to segment the tissue into hepatocytes (both malignant and non-malignant), stroma (fibrous tissue including the vasculature and bile ducts), and background/debris classes (Figure 1D), with subsequent segmentation (Figure 1E,F) of CD8+ cells via HALO^®^ Multiplex IHC algorithm (Indica Labs, USA).

We further processed the DIA outputs using a computational method based on hexagonal grid tiling as described by Rasmusson et al. [41] to extract CD8 spatial distribution indicators for malignant (HCC-stroma) and non-malignant (liver parenchyma-stroma) interfaces. In this study, we used a grid with hexagons that have a side length of 65 µm (Figure 1G) and aggregated the number of CD8+ cells and the area of tissue classes in each hexagon. Based on the abrupt change in the proportion of tissue class area across the grid, the hexagons that make up the edge between the stroma and the epithelium (which is either composed of benign hepatocytes or neoplastic HCC cells, depending on the annotation used) are identified (Figure 1G, yellow hexagons). The tiles are then ranked so that the hexagons on the extracted edge have rank 0 (Figure 1G, rank 0), the epithelial hexagons (HCC or liver) are assigned a positive rank equal to their distance from the nearest edge (Figure 1G, ranks 1 and 2), while the hexagons on the stromal side are assigned a negative rank equal to their distance from the edge (Figure 1G, ranks −1 and −2). Multiple combinations of the width of the central edge (TE) and analysis depth (number of ranks on both sides of TE) can be generated to provide different density characteristics. A five-hexagon wide interface zone having a three-rank wide central edge (Figure 1I,K) was used for further calculations in this study.

Indicators were extracted from the interface zones of the non-neoplastic liver (Figure 1H) and HCC (Figure 1J) to reflect the density profiles of CD8+ cells in both tissue compartments: the mean density (m) and standard deviation (sd) of CD8+ cells were calculated for the epithelial (T), stroma (S) and edge (TE) compartments, and indicators (center of mass (CM) and immunodrop (ID)) representing the whole IZ were also calculated. CM can represent either an increase in CD8+ density (when calculated using means) toward the epithelial aspect or a higher CD8+ variance (when using standard deviation) along the IZ. The ID indicator is a ratio between the mean CD8+ density in ranks −1 (stromal aspect) and 1 (epithelial aspect) and reflects an abrupt decrease in CD8 density across the TE, hence the ‘Immuno drop’.

### 2.4. Statistical Analysis and Modeling

Statistical analyses were performed using SAS software (version 9.4; SAS Institute Inc., Cary, NC, USA). A two-sided Welch t-test was applied for homogeneity of variance comparison. χ^2^ and Fisher’s exact test was used to examine the significant associations between categorical clinicopathological parameters. The nominal statistical significance level was set at *p* < 0.05. Since immune cell density distributions revealed left asymmetry, indicators were logarithm transformed for parametric statistics; however, for better readability, the prefix ‘log’ is not used in the text.

Cutoff Finder [43] was used to obtain an optimal cutoff value for each continuous indicator to test univariate OS predictions. The OS was estimated using the Kaplan–Meier method followed by log-rank testing to compare the statistical significance of the OS distributions. Multiple Cox regression was performed to further assess the independent prognostic value of the statistically significant biomarkers identified in the univariate analysis. Cox regression proportional hazards models were obtained using a stepwise likelihood ratio (LR) test to establish the independent prognostic significance of immunogradient indicators in the context of clinicopathological variables. Due to a limited cohort size, overfitting was minimized by leave-one-out cross-validation [44], and the most frequently selected variables were further tested in survival prediction models.

## 3. Results

### 3.1. Univariate Predictors of Overall Survival and Recurrence-Free Survival

**Figure 2:**
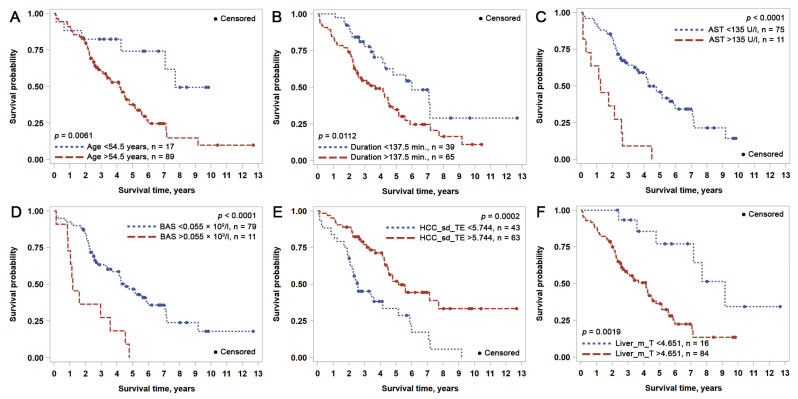
Kaplan-Meier overall survival (OS) plots for independent prognostic indicators identified by multiple Cox regression analysis: (**A**) patient age; (**B**) duration of surgery; (**C**) aspartate transaminase (AST); (**D**) peripheral blood basophil count; (**E**) standard deviation of CD8 density at the tumor edge (HCC_sd_TE); (**F**) mean CD8+ density within the epithelial aspect of the perineoplastic liver-stroma interface (Liver_m_T).

**Figure 3:**
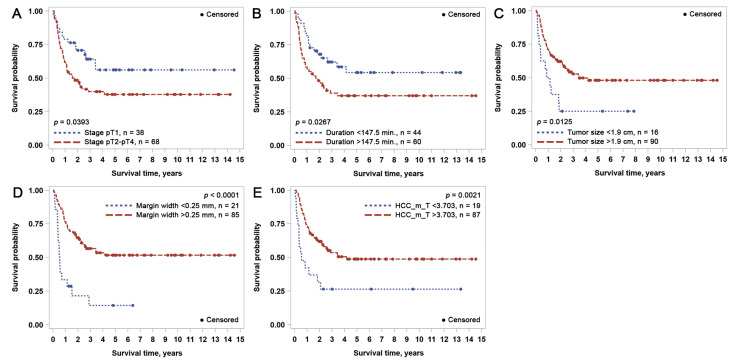
Kaplan-Meier recurrence-free survival (RFS) plots for independent prognostic indicators identified by multiple Cox regression analysis: (**A**) pT tumor stage; (**B**) duration of surgery; (**C**) tumor size; (**D**) tumor-free resection margin width; (**E**) mean CD8+ density in the epithelial aspect of tumor-stroma interface.

Table 3 contains the statistically significant results of the univariate regression analysis with the hazard ratio (HR) and log-rank test of the impact of clinicopathological characteristics and CD8+ cell spatial distribution indicators on OS and RFS. The OS and RFS probability plots for variables that also provided an independent prognostic impact (see Section 3.2) are presented in Figure 2 and Figure 3, respectively. Variables can be broadly grouped into three sets: conventional clinical and pathological parameters reported in daily practice, laboratory data obtained from blood analysis, and computed immunogradient indicators.

The statistically significant univariate predictors of worse OS were patient age > 54.5 years, stage pT2-pT4 (as opposed to pT1), any type of intravascular invasion, Ishak (modified Knodell) hepatitis activity index score > 5, >450 mL blood loss during liver surgery, longer (>137.5 min) duration of surgery, and longer (>30.5 days) hospital stay after surgery. Markedly elevated liver enzymes (ALT and AST > 135 U/L, GGT > 55 U/L), total bilirubin > 28.3 µmol/L and higher values of blood count variables (LEU > 8.89, NEU > 3.28, BAS > 0.055 × 10^9^/L) also had a negative impact on OS. Interface zone immunogradient indicators from both the malignant (HCC_CM_m, HCC_m_T, HCC_m_TE, HCC_sd_T, HCC_sd_TE—see Table 3) and non-malignant (Liver_CM_m, Liver_CM_sd, Liver_ID, Liver_m_T, Liver_sd_T—see Table 3) compartments demonstrated either a positive or negative effect on OS.

Statistically significant univariate predictors of shorter RFS were the advanced stage (pT2–pT4), longer (>147.5 min) duration of surgery, either R1 resection or a tumor-free margin < 0.25 mm, and, paradoxically, a smaller tumor size (<1.9 cm). Higher levels of liver enzymes (ALT > 22.5 U/L, AST > 36.5 U/L, ALP > 69 U/L), blood count variables (LEU > 6.585, NEU > 3.595, BAS > 0.0365 × 10^9^/L) and immunogradient indicators of the malignant compartment were also associated with a shorter RFS time in univariate analysis. None of the immunogradient indicators from non-malignant liver parenchyma was statistically significant for RFS.

### 3.2. Independent Predictors of Overall Survival and Recurrence-Free Survival

Independent prognostic features were explored using Cox regression models for OS and RFS using different sets of variables, starting from standard clinical and pathology data, and then supplementing them with laboratory and interface zone CD8+ cell profile data (Table 4).

Although Model 1 (LR: 22.3) revealed that older patients’ age and stage pT2–pT4 were the only independent predictors of worse OS, the addition of surgical data (Model 2) increased the prognostic power (LR: 29.6) by including the amount of blood lost during surgery. The addition of laboratory data (Model 3) further improved the prognostic value (LR: 46.18) taking into account the longer duration of surgery, intravascular invasion of the tumor, aspartate transaminase (AST) level > 135 U/L, and blood BAS count > 0.055 × 10^9^/L. Model 4 (LR: 61.5) was achieved by adding CD8+ cell density profiles at the HCC interface to model 3: a higher standard deviation of CD8+ density within the tumor edge improved OS (HR: 0.41, *p* = 0.0026). Adding CD8+ cell profile indicators from the non-malignant interface (Model 5, LR: 54.6) revealed mean CD8+ density in the epithelial aspect (i.e., remaining liver parenchyma) to be an independent predictor of worse survival.

Longer RFS could be predicted from a set of conventional independent features (Model 6, LR: 32.6): stage pT1, shorter duration of surgery (<147.5 min), a larger tumor size (>1.9 cm), wider resection margin around the tumor (>0.25 mm). Including CD8+ cell profiles at the malignant interface (Model 7, LR: 40.5) revealed the higher mean CD8+ density in the epithelial aspect (HCC) as an independent predictor of longer RFS.

### 3.3. Combined OS Prognostic Score

**Figure 4:**
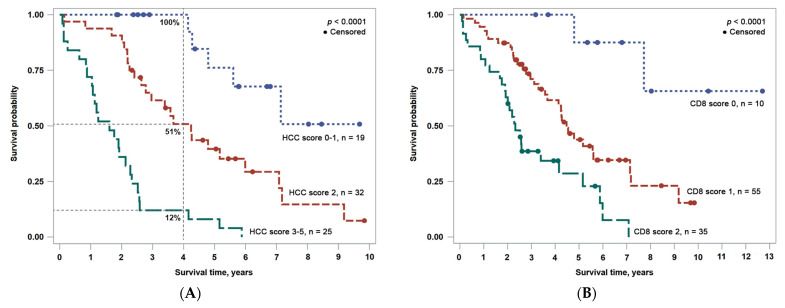
OS risk stratification based on combined prognostic scores: (**A**) comprehensive HCC OS score derived from five independent predictors from Model 5; (**B**) combined CD8 OS score derived from two independent predictors (CD8 density profiles at malignant and non-malignant interfaces).

Based on Model 5, a combined prognostic OS score was constructed by adding the contributions to OS from each independent variable (duration of surgery, aspartate transaminase (AST), blood basophil count (BAS), HCC_sd_TE, Liver_m_T): 1 for poor, 0 for good prognosis. Patients were assigned a prognostic category according to the combined score: scores 0–1 have low risk, score 2 has intermediate risk, and scores 3–5 make up the high-risk group, with a corresponding 5-year OS probability of 76%, 40%, and 8% (Figure 4A), respectively. The differences in OS probability between the groups were statistically significant: *p* < 0.0001 for low vs. high, *p* = 0.0045 for low vs. intermediate, and *p* < 0.0001 for intermediate vs. high risk. The prognostic power of the combined score is further illustrated by the probability of OS 4 years after liver resection at 100%, 51%, and 12% in the low, intermediate, and high-risk groups, respectively (Figure 4A). Of note, similar but less informative risk stratification could be achieved by combining only the impact of TILs and NILs (Figure 4B).

## 4. Discussion

Our study reveals that computational assessment of CD8+ cell density profiles at the interfaces of malignant and non-malignant epithelium with the surrounding stroma provides independent prognostic information for HCC patients after surgical liver resection. We also observed an independent impact of clinical (duration and/or blood loss during surgery) and laboratory (blood AST, basophils) parameters on the OS of HCC patients, which can further be combined into a comprehensive prognostic score. Meanwhile, only limited prognostic models could be obtained from conventional demographic (patient’s age) and pathology data (pT stage).

We applied hexagonal grid-based spatial analyses to assess CD8+ density profiles across automatically detected HCC-stroma and liver-stroma interfaces in surgical excision liver samples. The method enables the sampling of the interface zone ranks from which cell density-based indicators can be computed, including standard deviation (SD), as a measure of spatial heterogeneity of the densities along the interface. We found that a higher variance (SD) of mean CD8+ T lymphocyte density at the tumor edge of HCC is an independent predictor of longer OS. Recently, Krijgsman et al. [45] similarly reported that the high SD of the CD8+ T lymphocyte density distribution was the only DIA-derived parameter that provided an independent prediction of longer OS in a cohort of 236 Dutch patients with ER-positive breast cancer. The authors hypothesized that this phenomenon could be due to the positive contribution of local high-density CD8 infiltrates. Similar findings were recently published by Li et al. [46]: the presence of organized lymphoid aggregates (named tertiary lymphoid structures, which could be interpreted as a manifestation of local TIL density variance) in HCC was significantly associated with longer RFS, yet no effect on OS was observed. It is worthy of note that the precise location of the tertiary lymphoid structures in the tissue was not indicated in this study. Radziuviene et al. [33] also reported that the variance in CD8 density along the tumor edge is an independent predictor of OS in IHC HER2-borderline breast cancer without HER2 amplification; however, high CD8+ SD was associated with shorter OS in this patient cohort. The pathobiological and prognostic significance of irregular CD8 cell infiltrates along the tumor edge requires further elucidation; nevertheless, this adds to the accumulating evidence that intratumoral heterogeneity of a feature expression may be more informative than a general quantification of a feature per se [6,33,38,47,48].

On the other hand, we found that the high mean density of CD8+ cells in the epithelial aspect of the interface zone of the non-neoplastic liver parenchyma was an independent predictor of worse OS. The infiltration of cytotoxic CD8+ lymphocytes could reflect an active inflammatory process in the remaining liver. It is possible that in chronic HBV infection, virus-specific CD8+ cells acquire an exhaustive phenotype and produce fewer inflammatory cytokines [49], so a high CD8+ density may indicate ongoing liver damage driven by the immune system. No sufficient data on the current state of viral hepatitis was available in our cohort to test this hypothesis. In contrast, we demonstrate that higher mean CD8 density in the HCC tumor edge, most likely representing the host’s anti-tumoral immune response, is an independent predictor of longer RFS. Of note, this indicator was also significant for OS stratification in a univariate analysis (HCC_m_T with HR = 0.34, *p* = 0.0005), although it was not established as an independent predictor in multiple regression models for OS. Importantly, our computational assessment of CD8+ cell density profiles within the interfaces of malignant and non-malignant liver parenchyma with stroma revealed opposing prognostic effects of TILs and NILs, measurable in a surgical resection sample. Although a direct comparison cannot be made, contrary to Sun et al., who observed CD8+ density > 97 cells/mm^2^ in the center of the tumor to be an independent predictor of longer DFS and OS [8], in our study CD8+ cell densities obtained by DIA within the entire malignant, non-malignant, and stroma compartments did not reveal an independent prognostic impact on OS or RFS[REF].

Some features of the surgical procedure (duration and blood loss) revealed an independent prognostic value in our dataset. A longer duration of surgery was associated with worse OS (>137.5 min) and RFS (>147.5 min). An interpretation of this finding is not straightforward. One could speculate that multiple characteristics such as tumor size, multifocality, proximity to critical structures such as large intrahepatic vessels, etc. converge into a complex surgical setting that reflects the severity of liver disease in general. Lee et al. found that a longer duration (>210 min) of the liver resection procedure was associated with a higher proportion of patients with relapsed (RFS) and deceased (OS) HCC in 1-, 3- and 5-year periods [50]. Similarly, the duration of surgery was also found to be independently associated with an increased risk of severe complications after liver resection for colorectal cancer metastases [51]. However, in a recent study, He et al. [52] found no significant effect of the duration of surgery on OS in patients with HCC.

A negative effect of intraoperative blood loss has previously been reported on both RFS and OS in HCC patients [53,54]. Xiaocui Lv et al. [55] found that the extent of intraoperative blood loss during laparoscopic hepatectomy was directly associated with tumor size, surgery time, and type. Furthermore, increased intraoperative blood loss during the operation was found to be an independent predictor of HCC recurrence. Similarly, a longer hospital stay, which could reflect postoperative complications, was an independent predictor of hepatic decompensation after liver resection [56].

In our study, laboratory blood test data before surgery also contributed to the prognostic models. We found a negative impact of elevated alanine transaminase (ALT) and aspartate transaminase (AST) on OS, as previously reported [57]. Higher white blood cell, neutrophil, and basophil counts (Table 3) were associated with worse OS and RFS in our univariate analyses; these associations are less established in previous studies, in particular regarding RFS. A basophil count above 0.055 × 10^9^/L served as an independent predictor of worse OS in our cohort; Wu et al. [58] reported a similar finding in a subgroup of gastric cancer patients. In Wu’s study, a basophil count above 0.020 × 10^9^/L was an independent predictor of a lower overall response rate to anti-PD-1 immunotherapy plus chemotherapy combination as well as worse progression-free survival and OS. More studies are needed to clarify the role of increased basophil counts in the context of persistent viral infection and/or in tumor-host interaction [59].

Based on the independent prognostic features established in our study, we propose a comprehensive scoring system to stratify patients into three risk groups after HCC resection. In this model, aspartate transaminase (AST) and mean CD8 cell density in the epithelial aspect of the non-malignant interface zone reflect an active inflammatory process in the remaining liver, while the duration of surgery and the variance (SD) of the mean CD8+ T lymphocyte density at the tumor edge are tumor related. The peripheral blood basophil count is potentially a feature of the patient’s immune contexture, which could be affected by both antiviral and anti-tumoral responses. Similarly, the Barcelona Clinic Liver Cancer System (BCLC), which is the most widely used and validated staging system for HCC [60], incorporates the tumor parameters, residual liver function, and the general condition of the patient. BCLC classifies the patients into five groups: 0 (very early), A (early), B (intermediate), C (advanced), and D (terminal state).

The median OS time in the low (scores 0–1) risk group of our cohort was not reached, while it was approximately 50 months in the intermediate (score 2) group, and approximately 19 months in the high (scores 3–5) risk group. In a study by Wu et al. [61], the median survival time of the patients who underwent surgical resection in the BCLC 0-A (corresponding to low-risk) group was 52 months, it was 45 months in the BCLC B (intermediate risk) group, and it was 42 months in the BCLC C (high-risk) group, showing only marginal stratification.

Two years after the surgery, the low (scores 0–1), intermediate (score 2), and high (scores 3–5) risk groups of our cohort presented with OS rates of 100%, 91%, and 36%, respectively. In a validation study by Chan et al. [62], the 2-year probability of survival after curative treatment was 84% in BLCL group 0-A, 73% in BLCL group B, and 73% in BLCL group C. Two other staging systems were validated, the Cancer of the Liver Italian Program, CLIP (with a 2-year survival probability of 82% in the low-risk CLIP 0 group, 69% in intermediate-risk CLIP 1–3 group, and no patients in the high-risk CLIP 4–6 group) and the Chinese University Prognostic Index, CUPI (78% in low risk, 62% in the intermediate, and 100% in the high-risk group). The authors concluded that the performance of all three systems was suboptimal for the prediction of OS among patients who underwent curative HCC treatment [62]. Results of our study could help identify suitable indicators for a more precise prognostication system, although it is based on a retrospective single-center patient cohort of 106 patients and serves as a proof-of-concept that requires further validation studies

Our study has some additional limitations. First, we did not have sufficient data in our patient cohort to investigate prognostic models for disease specific survival, which would be relevant to further discriminate the impact of both neoplastic and non-neoplastic components of the liver disease. Second, although most of our analysis steps are automated, in some cases we could not achieve the satisfactory AI segmentation of malignant and non-malignant hepatocytes. The well-differentiated HCC, reactive atypia and dysplastic nodules were not properly distinguished in the WSI of IHC slides and, therefore, a pathologist manually annotated the neoplastic and nonneoplastic areas of interest in all resection samples.

## 5. Conclusions

In conclusion, we present computational models to assess CD8 density profiles in the interfaces of both malignant and non-malignant liver parenchyma with the surrounding stroma. In conjunction with clinical, pathological, and laboratory data, they enable comprehensive prognostic stratification of OS and RFS in patients with HCC after liver resection.

## Figures and Tables

**Table 1:** Clinical, pathological, and follow-up characteristics of the patient cohort.

Characteristic	Value
Patients	106 (100%)
Age, years	
Mean (range)	65 (13–82)
Median	64
Gender	
Male	82 (77.4%)
Female	24 (22.6%)
OS time, months	
Mean (range)	46 (1–152)
Median	39
Deceased	63 (59.4%)
RFS time, months	
Mean (range)	41 (1–174)
Median	25
Recurrences	56 (52.8%)
HCC grade	
G1	8 (7.5%)
G2	79 (74.5%)
G3	19 (18.0%)
pT stage	
T1	38 (35.9%)
T2	60 (56.6%)
T3	7 (6.6%)
T4	1 (0.9%)
Resection margin	
R0	86 (81.1%)
R1	20 (18.9%)
Largest tumor dimension, mm	
Mean (range)	48 (8–190)
Median	40
Surgical margin in R0 resections, mm	
Mean (range)	3.1 (0.1–25.0)
Median	3.1
History of viral infection	
HBV	9 (8.5%)
HCV	53 (50.0%)
None or unknown	44 (41.5%)
Hospitalization time, days	
Mean (range)	16 (4–70)
Median	13
Duration of surgery, min	
Mean (range)	170 (70–350)
Median	160

**Table 2:** Summary of the results of the preoperative blood test.

Variable	Mean	Median	Min	Max	N	No Data
LEU, ×10^9^/L	6.00	5.65	1.99	15.35	91	15
LYM, ×10^9^/L	2.94	1.62	0.22	106.00	90	16
MON, ×10^9^/L	0.56	0.50	0.12	1.38	90	16
EOS, ×10^9^/L	0.17	0.10	0.00	1.47	90	16
BAS, ×10^9^/L	0.03	0.02	0.00	0.20	90	16
RBC, ×10^12^/L	4.43	4.32	3.15	6.42	90	16
Albumin, g/L	40.25	40.30	24.80	51.10	64	42
Creatinine, µmol/L	74.05	71.00	42.00	144.00	82	24
Total bilirubin, µmol/L	17.75	14.45	5.10	52.80	82	24
Alanine transaminase (ALT), U/L	69.07	53.00	11.00	249.00	89	17
Aspartate transaminase (AST), U/L	69.35	56.00	19.00	209.00	86	20
Alkaline phosphatase (ALP), U/L	117.29	100.00	34.00	690.00	75	31
Gamma-glutamyl transferase (GGT), U/L	135.40	80.00	16.00	817.00	80	26
Alpha-fetoprotein (AFP), kU/L	669.54	10.65	0.50	30,000.00	94	12

**Table 3:** Univariate predictors of overall survival and recurrence-free survival.

Variable	HR	OS 95% CI	*p*-Value	HR	RFS 95% CI	*p*-Value
Conventional clinicopathological parameters						
Stage pT1	0.42	0.24–0.73	0.0023	0.54	0.30–0.98	0.0425
Age	3.14	1.33–7.42	0.0061	2.09	0.95–3.02	0.0697
Intravascular invasion present	2.13	1.28–3.54	0.0034	1.40	0.82–2.37	0.2137
Max tumor size	1.54	0.88–2.70	0.1300	0.45	0.24–0.86	0.0124
Ishak’s HAI score > 5	2.88	1.11–7.45	0.0292	1.96	0.86–4.45	0.1085
R1 resection	1.18	0.63–2.22	0.6073	3.52	1.95–6.38	<0.0001
Tumor-free margin width	0.79	0.43–1.46	0.4500	0.28	0.16–0.50	<0.0001
Blood loss during surgery	2.02	1.20–3.43	0.0074	0.45	0.14–1.43	0.1627
Duration of surgery	2.03	1.16–3.54	0.0112	1.87	1.07–3.29	0.0276
Duration of hospital stay	5.08	2.50–10.30	<0.0001	1.66	0.96–2.84	0.0647
Blood laboratory data						
Alanine transaminase (ALT)	4.29	1.33–7.74	<0.0001	2.92	1.05–8.10	0.0313
Aspartate transaminase (AST)	4.81	2.40–9.64	<0.0001	2.10	1.08–4.09	0.0263
Gamma-glutamyl transferase (GGT)	3.06	1.51–6.22	0.0012	1.69	0.79–3.62	0.1751
Alkaline phosphatase (ALP)	1.81	0.95–3.42	0.0660	3.69	1.14–11.94	0.0196
Total bilirubin	2.73	1.43–5.22	0.0015	1.65	0.93–2.91	0.0813
LEU count	2.50	1.11–5.63	0.0218	1.81	1.03–3.20	0.0376
NEU count	1.89	1.12–3.18	0.0145	2.27	1.29–4.01	0.0035
BAS count	3.67	1.86–7.23	0.0001	2.02	1.10–3.72	0.0206
Interface zone immunogradient indicators						
Malignant (HCC–stroma) interface *						
HCC_CM_m	0.49	0.26–0.95	0.0307	0.42	0.19–0.94	0.0291
HCC_m_T	0.34	0.18–0.64	0.0005	0.40	0.22–0.73	0.0021
HCC_m_TE	0.53	0.29–0.98	0.0397	0.55	0.32–0.93	0.0230
HCC_sd_T	0.60	0.37–1.00	0.0464	0.56	0.32–0.96	0.0328
HCC_sd_TE	0.39	0.24–0.65	0.0002	0.51	0.30–0.87	0.0113
Benign (liver–stroma) interface *						
Liver_CM_m	3.06	0.96–9.78	0.0475	1.73	0.62–4.78	0.2869
Liver_CM_sd	2.26	1.35–3.78	0.0016	0.38	0.12–1.21	0.0886
Liver_ID	0.57	0.34–0.96	0.0338	1.55	0.73–3.29	0.2486
Liver_m_T	3.65	1.54–8.67	0.0019	0.63	0.33–1.17	0.1374
Liver_sd_T	2.04	1.17–3.55	0.0104	0.71	0.42–1.22	0.2159

* Suffixes for immunogradient indicators (values are logarithm transformed for parametric statistics; for better readability, the prefix ‘log’ is not used): CM_m—the center of mass calculated from the mean CD8 density, CM_sd—center of mass calculated from the standard deviation of the CD8 density, m_T—mean CD8 density in the epithelial aspect of the interface zone, m_TE—mean CD8 density at the epithelial edge, sd_T—standard deviation of the CD8 density at the epithelial aspect of the interface zone, sd_TE—standard deviation of the CD8 density at the epithelial edge, ID—immunodrop.

**Table 4:** Independent predictors of OS and RFS based on multivariate Cox regression.

Variable	HR	95% CI	*p*-Value	χ^2^
OS Model 1: demographic and pathology data. LR: 22.30, *p* < 0.0001, N = 106				
Age > 54.5 years	3.93	1.59–9.69	0.0030	8.8142
Stage pT1 (versus T2–T4)	0.35	0.20–0.64	0.0007	11.6134
OS Model 2: demographic, pathology, and surgery data. LR: 29.60, *p* < 0.0001, N = 101				
Age > 54.5 years	4.46	1.76–11.33	0.0017	9.9012
Stage pT1 (versus T2–T4)	0.37	0.20–0.68	0.0013	10.2742
Blood loss during surgery > 450 mL	2.05	1.20–3.50	0.0090	6.8239
OS Model 3: demographic, pathology, surgery, and laboratory data. LR: 46.18, *p* < 0.0001, N = 81				
Age > 54.5 years	4.20	1.52–11.57	0.0055	7.7057
Duration of surgery > 137.5 min	2.61	1.42–4.81	0.0021	9.4252
Intravascular invasion present	3.10	1.71–5.61	0.0002	14.0066
Aspartate transaminase (AST) > 135 U/L	4.59	2.20–9.57	<0.0001	16.5728
Blood basophil (BAS) count > 0.055 × 10^9^/L	6.03	2.62–13.91	<0.0001	17.7860
OS Model 4: demographic, pathology, surgery, laboratory, and HCC (malignant) interface zone data. LR: 61.47, *p* < 0.0001, N = 81				
Age > 54.5 years	3.35	1.23–9.10	0.0177	5.6270
Duration of surgery > 137.5 min	2.07	1.09–3.92	0.0261	4.9488
Intravascular invasion present	3.00	1.65–5.43	0.0003	13.1234
Aspartate transaminase (AST) > 135 U/L	5.33	2.51–11.30	<0.0001	18.9716
Blood basophil (BAS) count > 0.055 × 10^9^/L	6.91	2.99–15.99	<0.0001	20.3752
HCC_sd_TE * > 5.744	0.41	0.23–0.73	0.0026	9.0769
OS Model 5: demographic, pathology, surgery, laboratory parameters, and data from both interface zones. LR: 54.61, *p* < 0.0001, N = 76				
Duration of surgery > 137.5 min	2.64	1.41–4.95	0.0023	9.2615
Aspartate transaminase (AST) > 135 U/L	4.50	2.01–10.11	0.0003	25.0296
Blood basophil (BAS) count > 0.055 × 10^9^/L	8.67	3.72–20.21	<0.0001	13.2883
HCC_sd_TE * > 5.744	0.33	0.18–0.59	0.0002	13.5590
Liver_m_T * > 4.651	4.81	1.73–13.28	0.0024	9.1963
RFS Model 6: demographic, pathology, and surgery data LR: 32.61, *p* < 0.0001, N = 104				
Stage pT1 (versus T2–T4)	0.40	0.20–0.82	0.0119	6.3248
Duration of surgery > 147.5 min	1.99	1.10–3.58	0.0224	5.2120
Max tumor size > 1.9 cm	0.20	0.09–0.44	<0.0001	15.6909
Tumor-free margin width > 0.25 mm	0.33	0.18–0.60	0.0003	12.8879
RFS Model 7: from demographic, pathology, surgery, and HCC (malignant) interface zone. LR: 40.50, *p* < 0.0001, N = 104				
Stage pT1 (versus T2–T4)	0.41	0.21–0.82	0.0108	6.4998
Duration of surgery > 147.5 min	2.07	1.13–3.80	0.0184	5.5614
Max tumor size > 1.9 cm	0.21	0.10–0.47	0.0001	14.7466
Tumor-free margin width > 0.25 mm	0.31	0.17–0.58	0.0002	13.5868
HCC_m_T * > 3.703	0.38	0.20–0.71	0.0024	9.2482

* Suffixes for immunogradient indicators (values are logarithm transformed for parametric statistics; for better readability, the prefix ‘log’ is not used): sd_TE—standard deviation of CD8 density at the epithelial edge, m_T—mean CD8 density at the epithelial aspect of IZ.

## Data Availability

The data presented in this study are available on request from the corresponding author. The data are not publicly available due to permit restrictions.

## Supplementary Materials

The following supporting information can be downloaded at: https://www.mdpi.com/article/10.3390/cancers15020366/s1, Figure S1. (**A**) CD8 Slide HIGH. (**B**) CD8 Slide LOW.
