# Supplementary figures and images for: Prognostic Value of CD8+ Lymphocytes in Hepatocellular Carcinoma and Perineoplastic Parenchyma Assessed by Interface Density Profiles in Liver Resection Samples
Source: Cancers (Basel). 2023 Jan 5;15(2):366. doi: 10.3390/cancers15020366 (PMC9857181; doi:10.3390/cancers15020366)

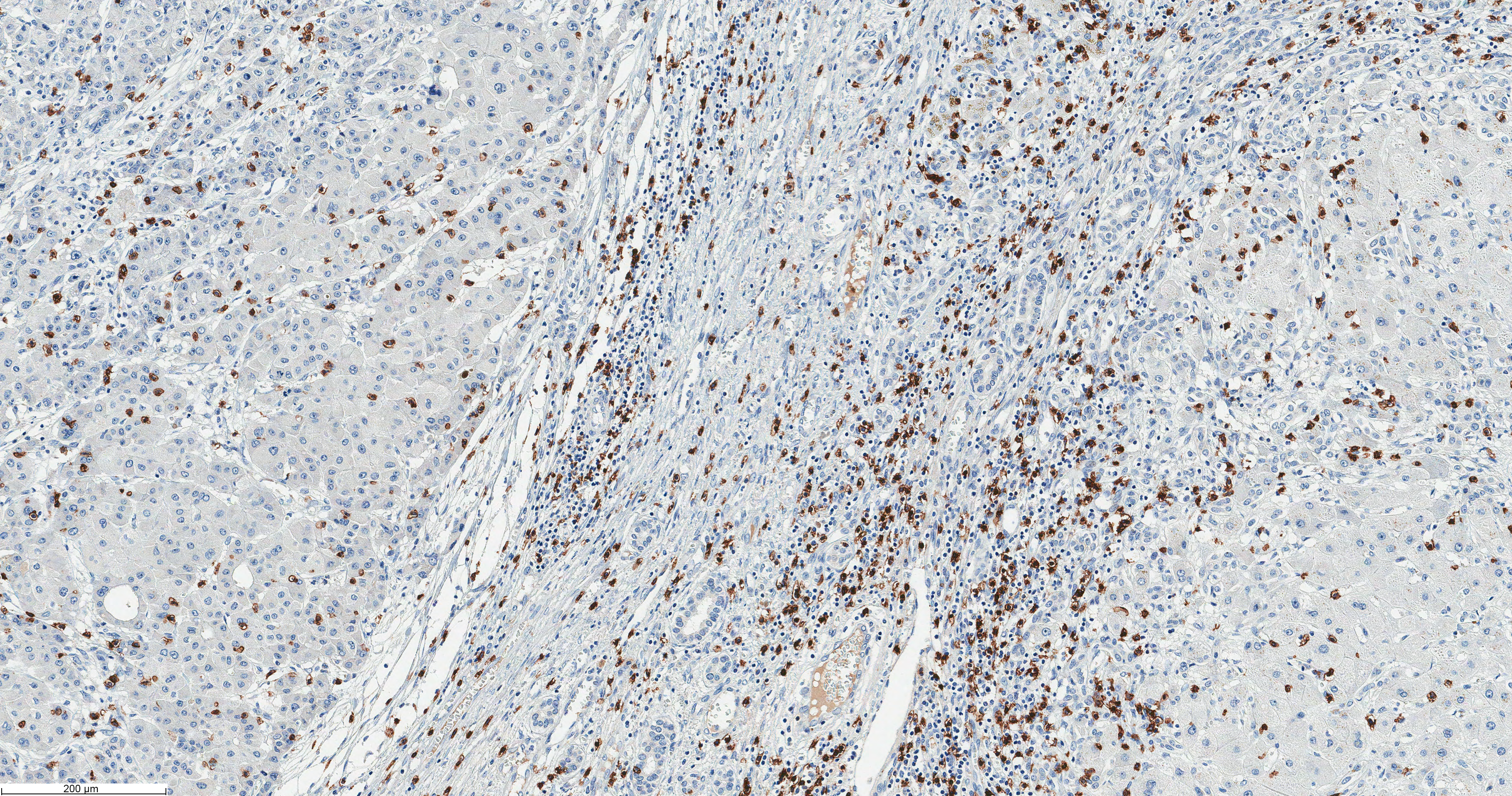

Supplement: Supplementary file 1 [file cancers-15-00366-s001.zip › SupplementaryFigS1A CD8 Slide HIGH (Revised 2023-01-05).tif]

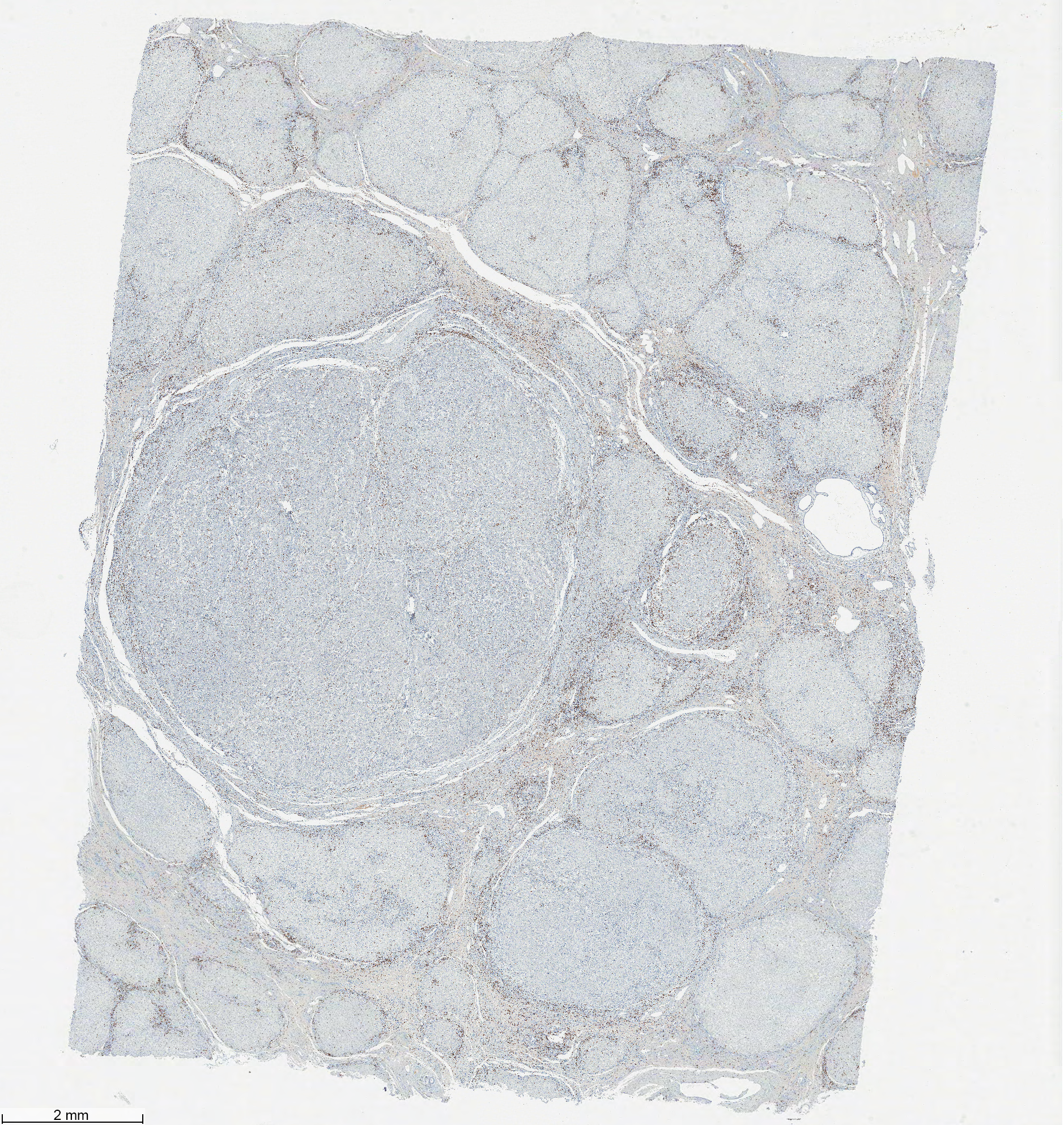

Supplement: Supplementary file 1 [file cancers-15-00366-s001.zip › SupplementaryFigS1B CD8 Slide LOW (Revised 2023-01-05).tif]
